# Design of Polarization-Independent Reflective Metalens in the Ultraviolet–Visible Wavelength Region

**DOI:** 10.3390/nano11051243

**Published:** 2021-05-08

**Authors:** Huifang Guo, Song Yue, Ran Wang, Yu Hou, Man Li, Kunpeng Zhang, Zichen Zhang

**Affiliations:** 1 Microelectronics Instruments and Equipment R&D Center, Institute of Microelectronics, Chinese Academy of Sciences, 3 Beitucheng West Road, Beijing 100029, China; guohuifang@ime.ac.cn (H.G.); wangran@ime.ac.cn (R.W.); houyu@ime.ac.cn (Y.H.); liman@ime.ac.cn (M.L.); zhangkunpeng@ime.ac.cn (K.Z.); 2 School of Microelectronics, University of Chinese Academy of Sciences, No. 19(A) Yuquan Road, Beijing 100049, China

**Keywords:** metasurface, reflective metalens, ultraviolet, silicon dioxide, aluminum

## Abstract

Flat lens or metalens, as one of the most important application branches of metasurfaces, has recently been attracting significant research interest. Various reflective and transmissive metalenses have been demonstrated in the terathertz, infrared and visible wavelength range. However, metalens operating in the ultraviolet (UV) wavelength range is rare. Moreover, the development of reflective UV metalens, the important counterpart of transmissive ones, falls far behind. In this work, with thorough investigation of material properties, we propose a reflective metalens based on silicon dioxide (SiO_2_) and aluminum (Al) that operates in the vacuum ultraviolet (VUV) to visible wavelength region. Four reflective metalenses were designed and optimized for wavelengths of 193, 441, 532 and 633 nm, and prominent focusing capability was observed, especially for the VUV wavelength of 193 nm. Dispersion characteristics of the metalenses were also studied within ±50 nm of the design wavelength, and negative dispersion was found for all cases. In addition, the SiO_2_ + Al platform can be, in principle, extended to the mid-infrared (IR) wavelength range. The reflective VUV metalens proposed in this work is expected to propel miniaturization and integration of UV optics.

## 1. Introduction

Miniaturization of optical systems plays an important role in modern information technology, enabling plenty of applications such as AR/VR [1], endoscopy [2], camera module of cell phones [3], miniaturized spectrometer [4], etc. Traditional optical systems manipulate wavefronts of light through accumulation of gradual phase change with the propagation of light waves, rendering optical components bulky, expensive and inefficient. Metasurfaces [5,6], delicate arrays of subwavelength optical scatterers/resonators, can generate abrupt local phase change at a 2D interface, providing a unique opportunity to planarize and miniaturize most traditional optical elements. Due to their great capability to manipulate multiple degrees of freedom of light, including amplitude, phase, polarization and wave vector, metasurfaces are believed to be able to revolutionize traditional optical components such as flat lenses [7,8], beam deflectors [9,10,11], wave plates [12,13], meta-holograms [14,15], sensors [16], perfect absorbers [17,18,19], orbital angular momentum generators [20,21,22], etc.

Among these, flat lens or metalens, as one of the most fundamental optical elements, has attracted significant research interest in recent years. Hitherto, on the one hand, various reflective and transmissive metalenses have been demonstrated in the terahertz (THz) [23,24], IR [25,26,27] and visible [28,29,30] wavelength range. On the other hand, however, metalens operating in the UV wavelength range is rare—despite this, UV optics have plenteous applications in the fields of high-resolution photolithography [31], imaging [32] and chemical/biological sensing [33]. Miniaturization of optical systems in the UV frequency range proved particularly challenging due to difficulties in material selection and fabrication of nanostructures with high aspect ratios for UV wavelengths. Metallic metasurfaces based on gold (Au) or silver (Ag) work well from THz to near-IR, but their performance deteriorates substantially in the visible to UV regions due to intrinsic Ohmic losses. Dielectric metalenses based on silicon (Si), titanium dioxide (TiO_2_), silicon nitride (Si_3_N_4_) and gallium nitride (GaN) have recently been demonstrated in the visible range. However, these devices suffer from the materials’ inter-band transitions (e.g., TiO_2_ at 3.2 eV and GaN at 3.4 eV), thus they could not cover the UV range. Very recently, Yao et al. demonstrated ultraviolet metasurfaces based on Si nanobars on quartz substrate working at a wavelength down to 290 nm [34]. Liu et al. demonstrated ultraviolet metasurfaces with ~80% efficiency based on Nb_2_O_5_ nanobricks on quartz substrate operating at the wavelength of 355 nm [35]. Zhang et al. achieved UV metalens operating at 325 nm and meta-hologram down to 266 nm wavelength, based on HfO_2_ metasurface on quartz substrate [36]. In the meantime, some theoretical works on UV metalens emerge as well, utilizing MgO [37] or Si_3_N_4_ [38] nanobricks on quartz (glass) substrates, respectively.

Despite the recent progress in transmissive UV metalens, it is noticed that the development of reflective UV metalens, the important counterpart of transmissive ones, falls far behind. Actually, reflective metalenses do exist in infrared and visible wavelength ranges [39,40], based on metal–insulator–metal (MIM) [40] and hybrid metal–dielectric configurations [41]. However, the working wavelength of reflective metalens can hardly be extended to the UV range, due to the absorption loss of metals and dielectrics involved in these works. Considering the importance of reflective UV metalens in applications such as photolithography and spectroscopy, we propose and demonstrate numerically in this work a polarization insensitive UV metalens operating in reflection mode. With a thorough investigation of material properties, we choose silicon dioxide (SiO_2_) and aluminum (Al) as the constituent material. Through arranging SiO_2_ nanopillars on top of an Al film, we propose a reflective metalens that pushes the working wavelength down to vacuum ultraviolet (VUV) range, i.e., 193 nm. The material combination chosen here not only holds superior UV property due to its large-enough bandgap and high-enough plasma frequency but also is cheap, abundant, CMOS compatible and easy to fabricate. Moreover, the material combination allows us to build reflective metalenses in the visible and even mid-IR wavelength range as well. The reflective VUV metalens proposed in this work holds promise to miniaturize UV optics, opening new pathways for various potential applications such as lithography and imaging, as well as spectroscopy.

## 2. Material Choice and Design of Structural Unit

**Figure 1:**
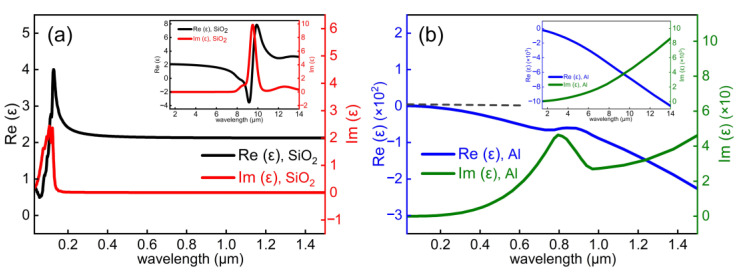
Real (Re(ε)) and imaginary (Im(ε)) part of dielectric constants of (**a**) SiO_2_ and (**b**) Al in the 0.03–1.5 µm range. Insets show their dielectric constants in the 1.5–14 µm range.

Reflective metalens based on MIM configuration usually has lower efficiency [40], thus we choose the hybrid metal-dielectric configuration to build our reflective metalens. To achieve a highly efficient reflective metalens in the targeted VUV wavelength region, optical properties of the constituent material, represented by its complex refractive index, need to be examined carefully. Simply speaking, the dielectric material used should have a negligible absorption loss, and the metal constituent should demonstrate metallic property in the whole operation wavelength range. Adhering to the above criteria, we select SiO_2_ as the dielectric and Al as the metal constituent. Dielectric constants of thermally grown SiO_2_ thin films are adapted from [42] and displayed in Figure 1a. Since the bandgap of thermally grown SiO_2_ thin film is quite large, i.e., 9.3 eV, corresponding to an interband transition wavelength of 133.3 nm, thermally grown SiO_2_ thin film is a good candidate for UV optics. Seen from Figure 1a, the imaginary part of dielectric constant of thermally grown SiO_2_ thin film is essentially zero down to 150 nm, covering the whole visible range and extending to the mid-IR (~8 μm). Meanwhile, compared to noble metals such as Au and Ag, Al has a higher plasma frequency and is a prominent plasmonic material in the UV range [43,44]. Seen from the dielectric constants of Al displayed in Figure 1b, Al keeps metallic (real part of dielectric constant *ε*_1_ < 0) for the whole wavelength range displayed here (0.03~14 μm). Based on the material properties, we foresee that the SiO_2_ + Al combination is a good platform to construct reflective metalens that works well from VUV (193 nm) all the way to the mid-IR (~8 μm) wavelength range.

**Figure 2:**
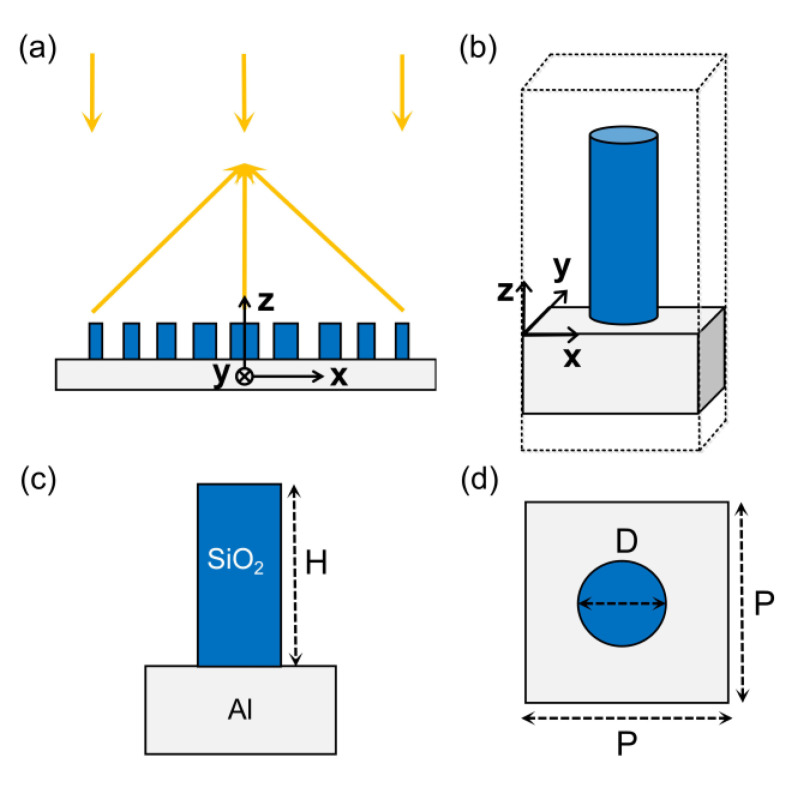
(**a**) Schematic of the reflective metalens. (**b**) Schematic of the unit cell: SiO_2_ nanopillar on Al film. (**c**,**d**) Side- and top-view of the unit cell showing the height H, diameter D and period P of SiO_2_ nanopillars.

While the SiO_2_ + Al material combination has the potential to build reflective metalenses from VUV to mid-IR range, we restrict ourselves in this work to the UV and visible wavelength range, with more attention devoted to the UV range. The operation principle and structural unit of the reflective metalens is schematically shown in Figure 2a,b, respectively. The structural unit is a SiO_2_ nanopillar on top of an Al film. Thickness of the Al film is set to a fixed value of 200 nm, which is thick enough to reflect all electromagnetic waves in the wavelength range considered in this work. SiO_2_ nanopillar is used as the phase shifter due to the high symmetry of its geometric shape, resulting in polarization insensitive focusing behavior. Figure 2c is a side view of the metalens unit, where H is the height of the SiO_2_ nanopillar. Figure 2d is a top view of the metalens unit, where P and D is the period and the diameter of the nanopillar, respectively. With plane wave incidence from above, the phase of reflected wave is controlled by changing the diameter D and height H of the nanopillars.

**Figure 3:**
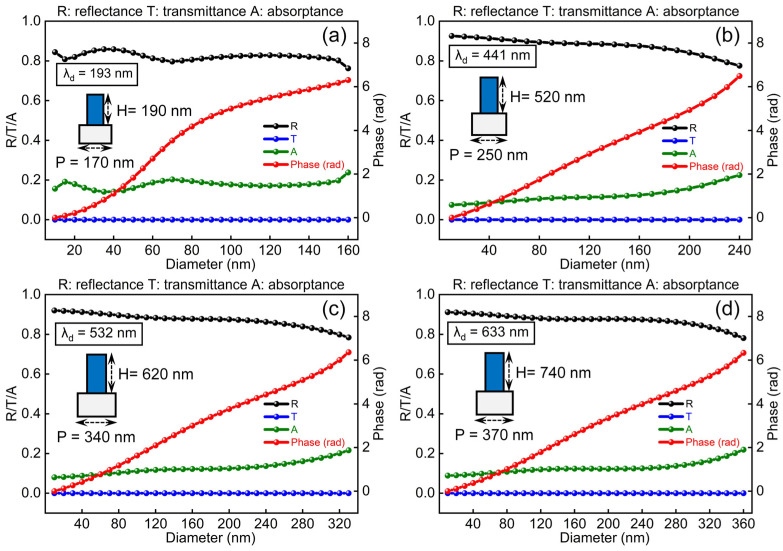
Simulated reflectance (R), transmittance (T), absorptance (A) and reflection phase of structural unit at respective design wavelength. (**a**) Design wavelength λ_d_ = 193 nm, optimal P = 170 nm and H = 190 nm. (**b**) λ_d_ = 441 nm, optimal P = 250 nm and H = 520 nm. (**c**) λ_d_ = 532 nm, optimal P = 340 nm and H = 620 nm. (**d**) λ_d_ = 633 nm, optimal P = 370 nm and H = 740 nm.

Numerical analysis of proposed metalenses was carried out using COMSOL Multiphysics, a commercial software based on finite element method. Considering that the calculation of three-dimensional metalens model requires a large amount of memory, and the cylindrical structure has polarization-independent characteristics, we simplified our model to a two-dimensional (2D) case (see Appendix A for the details of simulation set-up). In the following, all the simulation results were obtained from 2D models, thus the corresponding reflective metalens discussed is a cylindrical lens, whereas discussions over working wavelength and focusing property can be easily extended to the 3D case. Figure 3a–d show the simulated reflectance (R, black curves), transmittance (T, blue curves), absorptance (A, green curves) and reflection phase (red curves) of the structural units designed for typical wavelengths in the UV–visible range, i.e., *λ*_d_ = 193 nm, 441 nm, 532 nm and 633 nm, respectively. By optimizing geometric parameters of the SiO_2_ nanopillars at each design wavelength (see Appendix B for the details of optimization procedure), it is found that with the increase in diameter of the nanopillars, the unit cells can completely cover a phase change range of 0~2π while maintaining the reflectance close to 90%. The optimal period P of the unit cells and height H of SiO_2_ nanopillars for each design wavelength are labeled aside the insets in Figure 3a–d.

## 3. Reflective Metalens Device

Unit cells obtained above can provide local phase change at each position, and focusing functionality can be obtained by arranging unit cells according to the phase profile of a lens. The phase distribution φ(x) of a metalens should satisfy the following formula:

$$\varphi \left(x\right)=\frac{2\pi }{\lambda }\left(\sqrt{x^{2}+f^{2}}-f\right)$$

where *λ*, *x* and *f* are the incident wavelength, horizontal position from the center of the metalens and focal length of designed metalens, respectively. According to the phase distribution formula, the phase required at each position can be determined. Taking 193 nm design wavelength as an example, with optimized period and height of SiO_2_ nanopillars, only the diameter D of the nanopillars needs to be varied to achieve required phases. A home-written code was employed to match the diameter of nanopillars and the required phase at each position, and a corresponding metalens layout file was obtained, which was then imported into COMSOL to build the corresponding metalens model.

**Figure 4:**
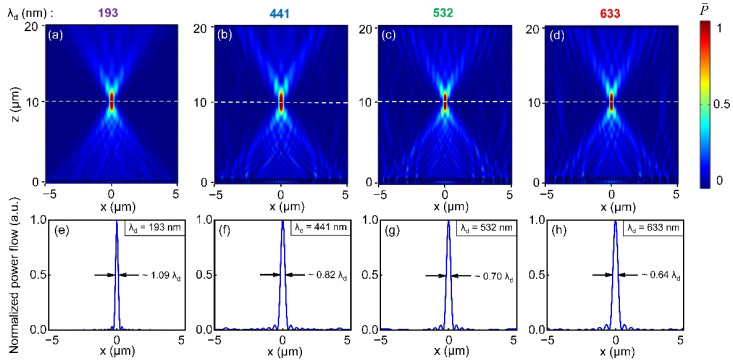
Focusing performance of reflective metalens designed for representative wavelengths in the UV–visible range. (**a**) λ_d_ = 193 nm. (**b**) λ_d_ = 441 nm. (**c**) λ_d_ = 532 nm. (**d**) λ_d_ = 633 nm. Normalized energy flux profiles along the white dashed lines at wavelengths of (**e**) 193 nm, (**f**) 441 nm, (**g**) 532 nm and (**h**) 633 nm. FWHMs of the focal spots are labelled on the plots.

Simulated reflected energy flux at typical wavelengths of 193, 441, 532 and 633 nm in the UV–visible range are shown in Figure 4a–d, respectively. As can be seen from Figure 4, all four reflective metalenses show a clear focal spot along the optical axis around 10 μm away from the Al surface, close to the design focal length of 10 μm. Due to the 2D model used in the simulations, the reflective metalenses demonstrated here are actually cylindrical lenses, and the focal spots are actually line focuses. However, the discussions over 2D reflective metalens can be easily extended to 3D cases. The designed focal length for all metalenses is 10 μm, and the actual focal length is 10.58, 11.28, 10.78 and 11.04 μm, respectively, as indicated by the dashed white lines in Figure 4a–d. Although there are small deviations between the actual focal length and the targeted value, focusing behavior is clearly seen for all four reflective metalenses in the UV–visible range. The reason for the deviation in focal length could be that the phase profile generated by the metasurface is an approximation to the perfect hyperbolic phase distribution of a lens, due to the pixel-wise discretization of the phase profile by metasurface. At the focus, the full-width half-maximum (FWHM) of the focal spots is also extracted and shown in Figure 4e–h. From extracted data, the FWHMs are 250 nm for operation wavelength of 193 nm, 406 nm for operation wavelength of 441 nm, 383 nm for operation wavelength of 532 nm and 510 nm for operation wavelength of 633 nm. Except for the case of VUV wavelength of 193 nm, all other three reflective metalenses realized sub-wavelength focusing. Although the VUV metalens does not achieve subwavelength focusing here, this is not inherent to the material choice. Instead, this is closely related to the numerical aperture (*N.A.*) of the metalenses. In principle, one can reduce the FWHM size for the VUV metalens through increasing its *N.A.*, simply by designing VUV metalens with shorter focal lengths, i.e., with a higher *N.A.* (see Appendix C for more discussions).

**Figure 5:**
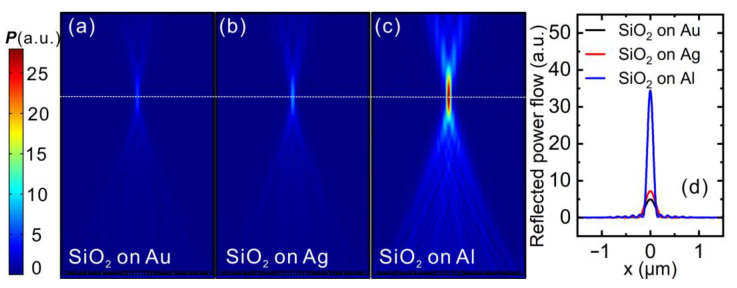
Focusing performance of reflective metalens designed for λ_d_ = 193 nm composed of (**a**) Au, (**b**) Ag and (**c**) Al mirror. Except for the material of metal component, all other parts are the same for the three cases. (**d**) Line cuts along x-direction across the focus.

It makes sense to have a direct comparison between SiO_2_ + Al metalens and SiO_2_ + Au/Ag metalens to confirm the superiority of metal Al in the VUV wavelength range. To do this, we replace the Al mirror in the metalens designed for 193 nm with Au and Ag, while keeping all other parts unchanged. Simulated reflected power flow are displayed in Figure 5a–c, which share the same color bar to the left. It is obvious that when the Al mirror is replaced by Au or Ag mirror, the focus, albeit still at the same position, becomes very dim. This denotes that the metalens device composed of Au or Ag mirror does not perform as well as that composed of Al mirror. This can be better seen if we plot a line-cut across the focus along x-direction, which is displayed in Figure 5d. It is seen again that the SiO_2_ + Al metalens outperforms the other two in the sense of focusing efficiency. This is understandable if one checks the optical properties of the Au, Ag and Al. Specifically, Au and Ag has interband transition starting from 2.4 and 4.02 eV [45], corresponding to a wavelength of 516.7 and 308.4 nm, respectively. Since the 193 nm falls within the interband transition absorption range of Au and Ag, metalens with Au and Ag mirror demonstrate heavy damping for incident light, resulting in reduced efficiency. Al, on the contrary, does not have interband transition absorption in the VUV wavelength considered here and has a higher bulk plasma frequency than Au and Ag [43,44], rendering it a better choice for reflective VUV metalens.

**Figure 6:**
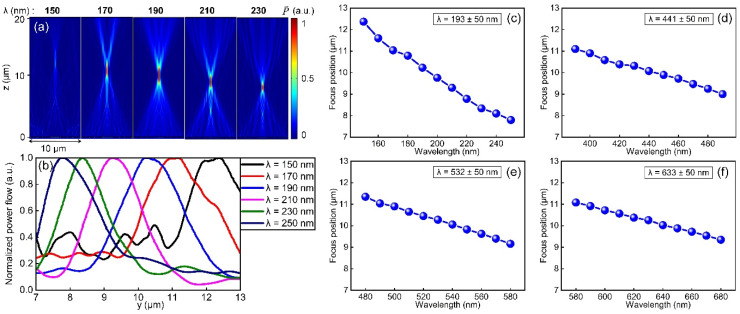
Dispersion characteristics of reflective metalenses. (**a**) Distribution of reflected energy flux for the metalens designed for 193 nm in the ±50 nm wavelength range. (**b**) Normalized reflected energy flux profiles around the focus along the optical axis in the wavelength range of 150 to 250 nm. (**c**) Extracted focal position of metalens as a function of incident wavelength for the design wavelength of 193 nm. (**d**–**f**), the same as (**c**), but for design wavelength of 441, 532 and 633 nm, respectively.

Dispersion characteristics of each metalens is also studied in the vicinity of ±50 nm around the designed target wavelength. Taking the metalens designed for 193 nm as an example, it can be seen from Figure 6a that the focusing effect of the reflective metalens is obvious except for the wavelength of 150 nm. Note that all subplots in Figure 6a share the same color bar on the right. A faint focus can be found if one looks closely at the subplot of λ = 150 nm, which indicates the lower wavelength limit of the reflective metalens optimized for 193 nm. On the one hand, 150 nm deviates a lot from the targeted wavelength of 193 nm; on the other hand, 150 nm approaches the absorption band-edge of thermally grown SiO_2_ thin films, leading to reduced efficiency of the focusing device. With the increase in operation wavelength, the focus becomes more obvious and moves continuously downward along the optical axis, meaning that the reflective metalens is negatively dispersive, in accordance with most metasurface devices [39,40]. Figure 6b shows the normalized reflected energy flux along the optical axis (*x* = 0) in the wavelength range of 150–250 nm. By tracking the peak positions of the energy flux under different wavelengths in Figure 6b, we obtained and plotted positions of focus of the reflective metalens designed for 193 nm under various incident wavelengths in Figure 6c. It can be seen that for the wavelength range of 150–250 nm, the actual focal position fluctuates within ±2 μm from the designed focal position and is negatively correlated with wavelength, demonstrating negative dispersion. Similar dispersion characteristics are observed for reflective metalenses designed for 441, 532 and 633 nm as well, as shown in Figure 6d–f, whereas the dispersion effect is weaker for these wavelengths compared to the case of 193 nm. Generally speaking, although dispersion behavior (chromatism) exists for our present reflective metalens design, which can be eliminated by adapting achromatic metalens design [46,47], the reflective metalens based on the combination of SiO_2_ nanopillars and Al film proves to work from the VUV to the visible wavelength range. Considering the potential of material property of SiO_2_ and Al (see Figure 1), the SiO_2_ + Al platform can be in principle extended to the mid-IR wavelength range, where one only needs to optimize the metalens design for the corresponding wavelength. For practical application of the reflective metalenses, one possible way could be the off-axis focusing method, i.e., deflecting the beam off from the optical axis while focusing it (see Appendix D for more details).

## 4. Conclusions

Through careful examination of the material property of the SiO_2_ + Al combination, a reflective cylindrical metalens based on SiO_2_ nanopillar on Al film was proposed and numerically demonstrated. Using COMSOL simulation and optimization, the diameter, height and period of unit cells of the SiO_2_ cylinder were determined. The optimized unit cells realize reflectance close to 90% while completely covering a 0~2π phase change. Four reflective metalenses were designed and optimized for wavelengths of 193, 441, 532 and 633 nm, and prominent focusing capability was observed for all reflective metalenses, especially for the VUV wavelength of 193 nm. Dispersion characteristics of the metalens were also studied within ±50 nm of the design wavelength. It is found that the actual focal position of the metalenses fluctuates within ±2 μm of the designed focal length and is negatively correlated with wavelengths, demonstrating negative dispersion. The material combination we used in this work not only allows us to build reflective metalenses in the UV to visible wavelength range but also holds the potential to be extended to the mid-IR. This makes the SiO_2_ + Al combination a versatile platform to build miniaturized optical systems based on reflective metasurface devices working in the UV all the way to the mid-IR, enabling a range of applications in fields such as high-resolution photolithography, chemical/biological sensing, imaging and spectroscopy, etc.

## Figures and Tables

**Figure A1:**
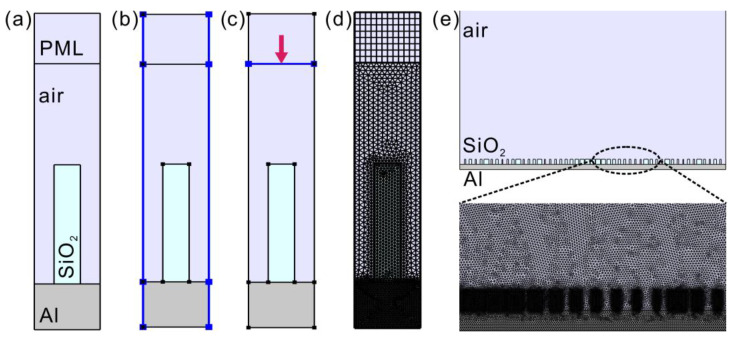
Simulation set-up showing (**a**) the Al, SiO_2_, air and PML domains, (**b**) periodic boundaries in x-direction, (**c**) the periodic launching and listening port and (**d**) the meshes used in the simulations. (**e**) Simulation set-up showing the whole metalens device and meshes of corresponding Al, SiO_2_ and air domains.

**Figure A2:**
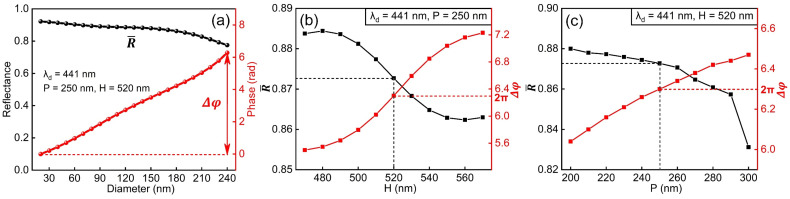
Parameter optimization procedure. (**a**) Reflectance and reflection phase of structural unit designed for 441 nm. Define the average reflectance as $\bar{R}$ and the phase coverage as Δφ, which will be used as the indicator for parameter optimization. (**b**) Selection of optimal parameter H with a preselected reasonable value of P. (**c**) Selection of optimal parameter P with the optimal value of H selected in the previous step.

**Figure A3:**
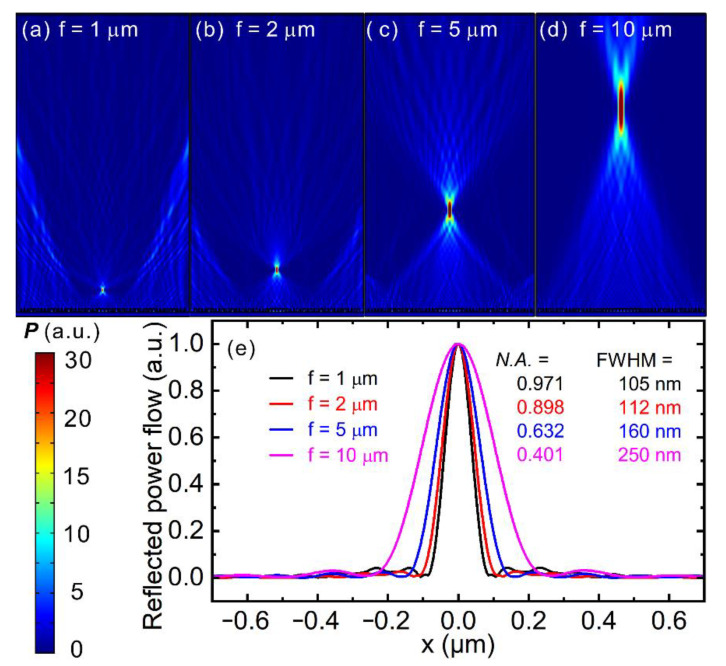
Simulated reflected power flow for a metalens designed for 193 nm wavelength with a focal length of (**a**) 1, (**b**) 2, (**c**) 5 and (**d**) 10 μm, respectively. Subplots (**a**–**d**) share the same color bar to the lower left. (**e**) Line cuts along the focus showing normalized power flow. It is obvious that with the increase in *N.A.*, the FWHM size of the focus decreases.

**Figure A4:**
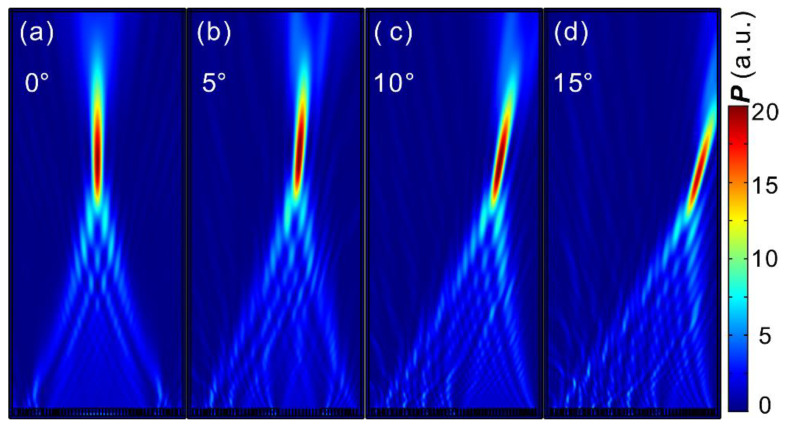
Simulated reflected power flow for a metalens designed for λ = 441 nm with a focal length of 20 μm and a deflection angle of (**a**) 0, (**b**) 5, (**c**) 10 and (**d**) 15°.

## Appendix A

### Details of Simulation Set-Up

Numerical analysis of metalenses was carried out using COMSOL Multiphysics, a commercial software based on finite element method (FEM). Considering that calculation of three-dimensional metalens model requires a huge amount of memory, and the cylindrical structure has polarization-independent characteristics, we simplified our model to a two-dimensional (2D) case. SiO_2_ and Al domains were assigned, as shown in Figure A1a, and the rest of the simulation domain is filled with air. A perfectly matched layer (PML) was added on the top of the unit cell, which absorbs reflected light from the structure. Floquet-periodic boundary conditions were used on both sides of the unit cell to simulate an infinite 1D array (Figure A1b), which is a generally adapted method in metasurface design [48]. Since the thickness of the Al mirror is several times thicker than the penetration depth of electromagnetic waves at the wavelengths considered in this work, transmittance of incident light is essentially zero. Thus, only one port is placed on the interior boundary of the PML, adjacent to the air domain (Figure A1c). This port serves as the launching and listening port at the same time, which is used to calculate reflectance and reflection phase of incident light through S-parameter calculations. Very fine mesh sizes were used, and maximal mesh sizes of 10 nm, λ/n_SiO_2_/20 and λ/20 were assigned to Al, SiO_2_ and air domains, respectively, where λ stands for the corresponding wavelength. The PML domain is meshed with a swept mesh method (Figure A1d). For the simulation of the whole metalens device, typical total number of mesh elements is around 4~7 × 10^5^, which requires roughly 10 GB of memory to run the simulation (Figure A1e).

## Appendix B

### Optimization Procedure of the Unit Cells of Meta-Atoms

Take the structure unit of metalens designed for 441 nm as an example. The main geometric parameters of the unit cell include period P, diameter D and height H of the SiO_2_ nanopillars. Thickness of the Al film is set to a fixed value of 200 nm, which is thick enough to reflect all electromagnetic waves in the wavelength range considered in this work. We generally started with a reasonable value of period P and scan the parameter H. For the selection of P, on the one hand, the period P should be smaller than the design wavelength to avoid higher-order diffraction. On the other hand, the period P cannot be too small, otherwise there would not be enough space for the diameter D of SiO_2_ nanopillars to vary. Thus, for the design wavelength of 441 nm, P = 250 nm is a moderate choice. Then, with the P value fixed, the height H is scanned. Under each value of parameter H, we varied the diameter D of the nanopillars and obtained one curve of reflectance and reflection phase (see Figure A2a). To facilitate the selection of optimal P and H values, the average reflectance of one curve is defined as $\bar{R}$, and the range of phase shift covered is defined as ∆*φ* (Figure A2a). We then used $\bar{R}$ and ∆*φ* as the indicator to seek for optimized values of P and H. The criterion is that the phase shift ∆*φ* should cover at least 0 to 2π, and the average reflectance $\bar{R}\;$ should be as high as possible. After a series of rough scan, the range of height H can be constrained to 470~570 nm. From Figure A2b, it can be found that when H is smaller than 520 nm, high reflectance can be achieved but the phase coverage is less than 2π. When H is greater than 520 nm, the phase coverage is over 2π but the reflectance decreases. Moreover, an increase in the height H of the nanopillar means increase in the aspect ratio of the structure, thereby increasing the difficulty of nano-fabrication. Therefore, an optimal H value of 520 nm can be picked up. Then, we fixed the H value and scanned parameter P within the range of ±50 nm around its initial value. From Figure A2c, it can be found that when P is less than 250 nm, high reflectance can be achieved but the phase coverage is less than 2π. When P is greater than 250 nm, the phase coverage is over 2π but the reflectance decreases obviously. Thus, it is finally determined that P = 250 nm and H = 520 nm is the best parameter combination for the design wavelength of 441 nm. The parameter optimization and selection procedure is similar for other design wavelengths.

## Appendix C

### Discussion over the FWHM Size of the Focus for the VUV Metalens

The FWHM of 250 nm for metalens designed for 193 nm seems discouraging; however, it is not inherent to the choice of materials. It is instead closely related to the numerical aperture (*N.A.*) of the metalens. Specifically, for the design wavelength of 193 nm, a focal length of 10 μm results in a *N.A.* of roughly 0.401 (through calculation of *N.A.* = *n*·sin θ, *n* = 1 for air, and sin θ can be calculated with the aperture size of the metalens and the focal length). The diffraction limit of such a metalens with *N.A.* = 0.401 can be calculated using d = λ/(2 *N.A.*), which is 240.6 nm. Thus, the simulated FWHM of 250 nm for the metalens with 10 μm focal length is close to the diffraction limit. One can, in principle, reduce the FWHM size of the focus through using metalenses with higher *N.A.* As a demonstration, we performed some numerical simulations of metalenses designed for 193 nm VUV wavelength, but with a focal length of 5, 2 and 1 μm instead (see Figure A3). In these cases, the *N.A.* is 0.632, 0.898 and 0.971 for focal length of 5, 2 and 1 μm, and the FWHM value of the focus is 160, 112 and 105 nm, respectively. These values are smaller than the value described in the main text and are close to their corresponding diffraction limit. Thus, the FWHM values can be improved by designing a VUV metalens with a shorter focal length, i.e., with a higher *N.A.* When *N.A.* is large enough, the VUV metalens can also achieve sub-wavelength focusing.

## Appendix D

### Possible Way to Implement Reflective Metalens in Practical Applications

Since the incident light and reflected light is on the same side of the reflective metalenses, special care needs to be taken for practical applications. One possible way we suggest is the off-axis focusing method, i.e., deflecting the beam off from the optical axis while focusing it. This can be accomplished by adding a linear phase gradient to the phase profile of the lens, so that the reflective metalens achieves beam deflection and focusing at the same time [48]. To demonstrate this, we plot in Figure A4 the reflected power flow for a metalens designed for λ = 441 nm with a focal length of 20 μm, which deflects the incident light to different angles while focusing them. The focal length used in the simulations is set to an affordable value, which is constrained by our computation resources. In principle, the focal length can be much larger so that the deflected focus can be completely separated from the incident beam, thus enabling implementation of reflective metalens in practical applications.

## References

[1] Lee G.-Y., Hong J.-Y., Hwang S., Moon S., Kang H., Jeon S., Kim H., Jeong J.-H., Lee B. (2018). Metasurface eyepiece for augmented reality. Nat. Commun..

[2] Pahlevaninezhad H., Khorasaninejad M., Huang Y.-W., Shi Z., Hariri L.P., Adams D.C., Ding V., Zhu A., Qiu C.-W., Capasso F. (2018). Nano-optic endoscope for high-resolution optical coherence tomography in vivo. Nat. Photonics.

[3] Yang S.-P., Seo Y.-H., Kim J.-B., Kim H., Jeong K.-H. (2019). Optical MEMS devices for compact 3D surface imaging cameras. Micro Nano Syst. Lett..

[4] Faraji-Dana M., Arbabi E., Arbabi A., Kamali S.M., Kwon H., Faraon A. (2018). Compact folded metasurface spectrometer. Nat. Commun..

[5] Yu N., Genevet P., Kats M.A., Aieta F., Tetienne J.P., Capasso F., Gaburro Z. (2011). Light Propagation with Phase Discontinuities: Generalized Laws of Reflection and Refraction. Science.

[6] Yu N., Capasso F. (2014). Flat optics with designer metasurfaces. Nat. Mater..

[7] Khorasaninejad M., Chen W.T., Devlin R.C., Oh J., Zhu A.Y., Capasso F. (2016). Metalenses at visible wavelengths: Diffraction-limited focusing and subwavelength resolution imaging. Science.

[8] Bai W., Yang P., Wang S., Huang J., Chen D., Zhang Z., Yang J., Xu B. (2019). Tunable Duplex Metalens Based on Phase-Change Materials in Communication Range. Nanomaterials.

[9] Kita S., Takata K., Ono M., Nozaki K., Kuramochi E., Takeda K., Notomi M. (2017). Coherent control of high efficiency metasurface beam deflectors with a back partial reflector. APL Photonics.

[10] Li N., Fu Y.H., Dong Y., Hu T., Xu Z., Zhong Q., Li D., Lai K.H., Zhu S., Lin Q. (2019). Large-area pixelated metasurface beam deflector on a 12-inch glass wafer for random point generation. Nanophotonics.

[11] Hong X., Feng S., Guo H., Li C. (2019). A beam deflector with dielectric metasurfaces in the terahertz region. Laser Phys..

[12] Zhang X., Kong D., Yuan Y., Mei S., Wang L., Wang G. (2020). Broadband and dispersion-free reflective silver metasurfaces as half-wave plate and vortex-beam generator. Opt. Commun..

[13] Maiolo L., Ferraro A., Maita F., Beccherelli R., Kriezis E.E., Yioultsis T.V., Zografopoulos D.C. (2019). Quarter-wave plate metasurfaces on electromagnetically thin polyimide substrates. Appl. Phys. Lett..

[14] Ren H., Fang X., Jang J., Burger J., Rho J., Maier S.A. (2020). Complex-amplitude metasurface-based orbital angular momentum holography in momentum space. Nat. Nanotechnol..

[15] Wang L., Zhang W., Yin H., Zhang X. (2019). Ultrasmall Optical Vortex Knots Generated by Spin-Selective Metasurface Holograms. Adv. Opt. Mater..

[16] Wang Y., Zhao C., Wang J., Luo X., Xie L., Zhan S., Kim J., Wang X., Liu X., Ying Y. (2021). Wearable plasmonic-metasurface sensor for noninvasive and universal molecular fingerprint detection on biointerfaces. Sci. Adv..

[17] Mou N., Sun S., Dong H., Dong S., He Q., Zhou L., Zhang L. (2018). Hybridization-induced broadband terahertz wave absorption with graphene metasurfaces. Opt. Express.

[18] Swett D.W. (2020). Near Zero Index Perfect Metasurface Absorber using Inverted Conformal Mapping. Sci. Rep..

[19] Yue S., Hou M., Wang R., Guo H., Hou Y., Li M., Zhang Z., Wang Y., Zhang Z. (2020). Ultra-broadband metamaterial absorber from ultraviolet to long-wave infrared based on CMOS-compatible materials. Opt. Express.

[20] Tang S., Li X., Pan W., Zhou J., Jiang T., Ding F. (2019). High-efficiency broadband vortex beam generator based on transmissive metasurface. Opt. Express.

[21] Zhao A., Pham A., Drezet A. (2021). Plasmonic fork-shaped hologram for vortex-beam generation and separation. Opt. Lett..

[22] Ren H., Briere G., Fang X., Ni P., Sawant R., Heron S., Chenot S., Vezian S., Damilano B., Brandli V. (2019). Metasurface orbital angular momentum holography. Nat. Commun..

[23] Kargar R., Rouhi K., Abdolali A. (2020). Reprogrammable multifocal THz metalens based on metal–insulator transition of VO2-assisted digital metasurface. Opt. Commun..

[24] Zhao F., Li Z., Dai X., Liao X., Li S., Cao J., Shang Z., Zhang Z., Liang G., Chen G. (2020). Broadband Achromatic Sub-Diffraction Focusing by an Amplitude-Modulated Terahertz Metalens. Adv. Opt. Mater..

[25] Tanriover I., Demir H.V. (2019). Broad-band polarization-insensitive all-dielectric metalens enabled by intentional off-resonance waveguiding at mid-wave infrared. Appl. Phys. Lett..

[26] Fan Q., Liu M., Yang C., Yu L., Yan F., Xu T. (2018). A high numerical aperture, polarization-insensitive metalens for long-wavelength infrared imaging. Appl. Phys. Lett..

[27] Engelberg J., Zhou C., Mazurski N., Bar-David J., Kristensen A., Levy U. (2020). Near-IR wide-field-of-view Huygens metalens for outdoor imaging applications. Nanophotonics.

[28] Abdollahramezani S., Chizari A., Dorche A.E., Jamali M.V., Salehi J.A. (2017). Dielectric metasurfaces solve differential and integro-differential equations. Opt. Lett..

[29] Fan Z.B., Qiu H.Y., Zhang H.L., Pang X.N., Zhou L.D., Liu L., Ren H., Wang Q.H., Dong J.W. (2019). A broadband achromatic metalens array for integral imaging in the visible. Light Sci. Appl..

[30] Park J.S., Zhang S., She A., Chen W.T., Lin P., Yousef K.M.A., Cheng J.X., Capasso F. (2019). All-Glass, Large Metalens at Visible Wavelength Using Deep-Ultraviolet Projection Lithography. Nano Lett..

[31] Naulleau P., Andrews D.L., Lipson R.H., Nann T. (2019). 2.17-Optical Lithography. Comprehensive Nanoscience and Nanotechnology.

[32] Day R.N., Davidson M.W. (2009). The fluorescent protein palette: Tools for cellular imaging. Chem. Soc. Rev..

[33] Greenfield N.J. (2006). Using circular dichroism spectra to estimate protein secondary structure. Nat. Protoc..

[34] Deng Y., Wang X., Gong Z., Dong K., Lou S., Pégard N., Tom K.B., Yang F., You Z., Waller L. (2018). All-Silicon Broadband Ultraviolet Metasurfaces. Adv. Mater..

[35] Huang K., Deng J., Leong H.S., Yap S.L.K., Yang R.B., Teng J., Liu H. (2019). Ultraviolet Metasurfaces of ≈80% Efficiency with Antiferromagnetic Resonances for Optical Vectorial Anti-Counterfeiting. Laser Photonics Rev..

[36] Zhang C., Divitt S., Fan Q., Zhu W., Agrawal A., Lu Y., Xu T., Lezec H.J. (2020). Low-loss metasurface optics down to the deep ultraviolet region. Light Sci. Appl..

[37] Ali F., Aksu S. (2021). A hybrid broadband metalens operating at ultraviolet frequencies. Sci. Rep..

[38] Kanwal S., Wen J., Yu B., Kumar D., Chen X., Kang Y., Bai C., Zhang D. (2020). High-Efficiency, Broadband, Near Diffraction-Limited, Dielectric Metalens in Ultraviolet Spectrum. Nanomaterials.

[39] Yang H., Li G., Su X., Cao G., Zhao Z., Chen X., Lu W. (2017). Reflective metalens with sub-diffraction-limited and multifunctional focusing. Sci. Rep..

[40] Zhang Y., Yang B., Liu Z., Fu Y. (2020). Polarization Controlled Dual Functional Reflective Planar Metalens in Near Infrared Regime. Coatings.

[41] Tang F., Ye X., Li Q., Li H., Yu H., Wu W., Li B., Zheng W. (2020). Quadratic Meta-Reflectors Made of HfO_2_ Nanopillars with a Large Field of View at Infrared Wavelengths. Nanomaterials.

[42] Rodríguez-de Marcos L.V., Larruquert J.I., Méndez J.A., Aznárez J.A. (2016). Self-consistent optical constants of SiO_2_ and Ta_2_O_5_ films. Opt. Mater. Express.

[43] Knight M.W., King N.S., Liu L., Everitt H.O., Nordlander P., Halas N.J. (2014). Aluminum for Plasmonics. ACS Nano.

[44] McMahon J.M., Schatz G.C., Gray S.K. (2013). Plasmonics in the ultraviolet with the poor metals Al, Ga, In, Sn, Tl, Pb, and Bi. Phys. Chem. Chem. Phys..

[45] Johnson P.B., Christy R.W. (1972). Optical Constants of the Noble Metals. Phys. Rev. B.

[46] Shrestha S., Overvig A.C., Lu M., Stein A., Yu N. (2018). Broadband achromatic dielectric metalenses. Light Sci. Appl..

[47] Zang W., Yuan Q., Chen R., Li L., Li T., Zou X., Zheng G., Chen Z., Wang S., Wang Z. (2020). Chromatic Dispersion Manipulation Based on Metalenses. Adv. Mater..

[48] Arbabi A., Horie Y., Bagheri M., Faraon A. (2015). Dielectric metasurfaces for complete control of phase and polarization with subwavelength spatial resolution and high transmission. Nat. Nanotechnol..

